# Cross-modal metaphorical mapping of spoken emotion words onto vertical space

**DOI:** 10.3389/fpsyg.2015.01205

**Published:** 2015-08-11

**Authors:** Pedro R. Montoro, María José Contreras, María Rosa Elosúa, Fernando Marmolejo-Ramos

**Affiliations:** ^1^Departamento de Psicología Básica I, Facultad de Psicología, Universidad Nacional de Educación a DistanciaMadrid, Spain; ^2^Gösta Ekman Laboratory, Department of Psychology, Stockholm UniversityStockholm, Sweden

**Keywords:** emotions, vertical space, cross-modal procedure, embodiment, metaphorical mapping

## Abstract

From the field of embodied cognition, previous studies have reported evidence of metaphorical mapping of emotion concepts onto a vertical spatial axis. Most of the work on this topic has used visual words as the typical experimental stimuli. However, to our knowledge, no previous study has examined the association between affect and vertical space using a cross-modal procedure. The current research is a first step toward the study of the metaphorical mapping of emotions onto vertical space by means of an auditory to visual cross-modal paradigm. In the present study, we examined whether auditory words with an emotional valence can interact with the vertical visual space according to a ‘positive-up/negative-down’ embodied metaphor. The general method consisted in the presentation of a spoken word denoting a positive/negative emotion prior to the spatial localization of a visual target in an upper or lower position. In Experiment 1, the spoken words were passively heard by the participants and no reliable interaction between emotion concepts and bodily simulated space was found. In contrast, Experiment 2 required more active listening of the auditory stimuli. A metaphorical mapping of affect and space was evident but limited to the participants engaged in an emotion-focused task. Our results suggest that the association of affective valence and vertical space is not activated automatically during speech processing since an explicit semantic and/or emotional evaluation of the emotionally valenced stimuli was necessary to obtain an embodied effect. The results are discussed within the framework of the embodiment hypothesis.

## Introduction

Emotion concepts have been researched extensively, particularly in relation to abstract and concrete concepts (see Altarriba and Bauer, 2004), and have become a topic of particular interest in the embodied cognition framework (e.g., Niedenthal et al., 2005, 2009; see also Meteyard et al., 2012). Specifically, it has been argued that abstract and emotion concepts have sensorimotor properties much like concrete concepts. For example, it has been shown that there is a metaphorical association between emotionally valenced concepts and the vertical plane (e.g., Meier and Robinson, 2004; Meier et al., 2011; Sasaki et al., 2012, 2015; Santiago et al., 2012; Marmolejo-Ramos and Dunn, 2013; Marmolejo-Ramos et al., 2013, 2014; Xie et al., 2014, 2015; Damjanovic and Santiago, 2015). Most of the work on the association between emotion words and the vertical axis has used visual words as typical experimental stimuli. Indeed, most tasks rely on within-modal tasks (e.g., effects of images on word processing), chiefly visual tasks, and to a certain extent rarely examine cross-modal effects (e.g., effects of sounds on word processing); let alone study emotions in cross-modal processing (see Gerdes et al., 2014). This study aims to investigate the association between emotional auditory stimuli and vertical visual space. Specifically, an auditory to visual cross-modal paradigm task is used to explore the limits of the metaphorical mapping between concepts and bodily simulated space. That is, we studied whether auditory words with an affective valence can influence the spatial localization of visual stimuli in line with the ‘positive-up/negative-down’ vertical spatial metaphor. Additionally, we included different intervals between the auditory word and visual cue in order to explore the automaticity (or lack thereof) of this audiovisual emotional processing.

The introduction in this article is divided as follows. Firstly, some examples of research in the embodiment of emotions are presented to outline the overarching topic of the studies reported in this article. Secondly, the specific case of conceptual metaphors and emotions is considered. In particular, research on the valence-space metaphor is discussed since it constitutes the exact scope of this article. Finally, research relating to the timing of the valence-space metaphor is examined. This is a new aspect, which is currently being investigated.

### Embodiment of Emotions

Traditionally, most research on emotions has employed visual stimuli. However, recent work has used non-visual emotional stimuli in relation to other modalities. In a study by Tajadura-Jiménez et al. (2010), it was shown that unpleasant approaching sounds elicit a more intense emotional response than pleasant or neutral preceding sounds. The emotional response of the participants consisted of a greater self-reported emotional experience and a greater facial muscle response during unpleasant approaching sounds than during the preceding conditions. Furthermore, listening to white noise, a type of sound rated as unpleasant, while people provided odor ratings for different smells, led to lower pleasantness and sweetness and higher dryness odor ratings (Velasco et al., 2014). Other studies using emotionally valenced tactile stimuli have found that even touch gestures communicated remotely (i.e., via a tactile device) can convey different emotional intentions (Rantala et al., 2013). Specifically, Rantala et al. (2013) found that squeeze actions are associated with unpleasant and aroused emotional intentions, whereas finger touch was better at conveying pleasant and relaxed emotional intentions. These studies suggest that emotional stimuli can indeed influence the body’s somatosensory and sensorimotor systems and emotions can indeed be conveyed through these systems.

Research has shown that emotions are subserved by somatosensory and sensorimotor systems that work together in response to internal or external stimulus events (e.g., Scherer, 2005). That is, emotions are made up of interoceptions, exteroceptions, and memories that are instantiated whenever an emotion is re-experienced, recalled or evoked (see Niedenthal et al., 2005, 2009). Indeed, recent neuroimaging research further indicates that experiencing emotions entails the activation of distributed multimodal brain networks involved in various psychological processes (Oosterwijk et al., 2012). Further, research on the embodiment of emotions has shown that even emotion concepts and words can elicit the activation of such multimodal networks (see Niedenthal et al., 2005, 2009; see also Marmolejo-Ramos et al., 2015). For example, Wilson-Mendenhall et al. (2013) found that the sensorimotor cortex, the amygdala and the hippocampus were activated during vivid imagination of situations referring to physical danger and social evaluations^1^ (where sensorimotor activations may have resulted from the simulation *per se* rather than the emotional activation). As the authors indicated, the hippocampus is involved in other psychological processes such as binding multimodal mnemonic information and simulating future and imagined situations. Such findings support the idea that multimodal simulations are deployed when emotions are processed. Recent research on the conceptual modality-switching cost effect (see Pecher et al., 2003) lends further support to the multimodality associated to emotions. Specifically, it has been found that there is a switching cost when shifting from somatosensory to emotional modality but not the other way around (Dagaev and Terushkina, 2014). The authors further argued that activation of emotion concepts entails the activation of somatosensory modalities such as touch, pressure, vibration, temperature, pain, and joint and muscle sensitivity. Overall, these studies indicate that the processing of emotion concepts entails the activation of concepts relating to somatosensory and sensorimotor systems. However, research investigating how emotions are transferred across somatosensory and sensorimotor modalities in real modality-switching tasks is just emerging (see Velasco et al., 2014). Specifically, it would be important to investigate what emotions are more or less easily transferred across somatosensory and sensorimotor systems and what their time course is during switching tasks.

### Conceptual Metaphors and Emotions

Conceptual metaphors occur when concepts are mapped from a source domain onto a target domain. Specifically, target domains refer to abstract concepts that are to be mapped onto source domains that are concrete and bodily based (see Gallese and Lakoff, 2005). Based on these premises, Wilson and Gibbs (2007) found that performing body actions, or even imagining them, facilitated the comprehension of metaphorical sentences, when compared to not performing any sort of action. In the specific case of emotions and space, emotions (target domain) are conceptualized as spatial locations (source domain). That is, just as upper locations tend be evaluated as more positive than lower spatial locations, positive words tend to be allocated in upper spatial areas, while negative words tend to be allocated in lower areas (Marmolejo-Ramos et al., 2013). According to Lakoff (2014), metaphorical mappings rely on the human body itself and its neurological substratum as the source domain in order to represent physical properties (e.g., motion and space). That is, the sensorimotor and somatosensory systems dictate the experience with the physical environment used to ground abstract concepts (e.g., these systems enable the processing of physical properties like high-low as analogs of valenced abstract concepts). Thus, the metaphorical mapping of emotions onto space requires somatosensory and sensorimotor simulations in order to comprehend the linkage from the target to the source domain.

In order to test the metaphorical mapping of emotions onto space, Ansorge and Bohner (2013; Ansorge et al., 2013) used an implicit association task in which participants categorized words like ‘up’ as elevated or less elevated and affective words like ‘happy’ as positive or negative. Interestingly, when spatial and affective stimuli were metaphorically congruent and required the same response (e.g., up-happy), faster responses and fewer errors were found than when spatial and affective stimuli were incongruent and required a different response (see Experiment 1). That is, faster responses were observed when target words were presented in spatial congruent locations (e.g., ‘happy’ in the upper part of a computer screen) than when they were presented in incongruent locations (e.g., ‘sad’ in the upper part of a computer screen) and this association was seemingly implicit. Other studies confirmed that there is even a mapping of emotion sentences onto space; however, such an association holds only when the task demands an explicit affective evaluation of the target (Marmolejo-Ramos et al., 2014; Experiment 2). In the case of emotion words, it has been shown that such an association occurs only when valence is to be explicitly evaluated or when they refer to emotional states that have discernible body postures (Dudschig et al., 2015). Note that these types of results are informative as to within-modal emotion processing.

However, not much is known as to multimodal and cross-modal emotion processing. Indeed, the employment of cross-modal paradigms for the study of conceptual–physical interactions could contribute relevant data to decide between alternative models of embodiment. Recently, Meteyard et al. (2012) reviewed four different theories of embodiment arranged in a continuum from “strong embodiment” (complete dependence on the relationship to sensory-motor systems) to completely “unembodied” (complete independence between both). The evidence revised by Meteyard et al. (2012) supports balanced/moderate versions of the embodiment hypothesis, which propose that sensory and motor information is activated when a semantic representation is accessed. In the present study, we test the hypothesis that deep semantic processing is needed to display the effect of embodiment. To prove this, two experiments were planned; in Experiment 1 only shallow processing was required, while in Experiment 2, the effect of emotional versus non-emotional processing was contrasted.

A review paper by Gerdes et al. (2014), noted that there is behavioral, physiological, and electrophysiological evidence showing the effects that emotional visual stimuli have on auditory processing. However, only a couple of studies have investigated how emotional sounds influence visual processing (see also Table 1 in Gerdes et al., 2014). In one of these studies, it was found that when emotionally valenced stimuli were visually presented, recognition of visually presented neutral stimuli was impaired. However, when emotionally valenced stimuli were auditorily presented, recognition of visually presented neutral stimuli was enhanced (Zeelenberg and Bocanegra, 2010). Furthermore, another study found behavioral effects of emotional sounds on visual processing only when visual items were presented on the right visual hemifield^2^ (Harrison and Davies, 2013; see Brosch et al., 2008 for a study in which emotionally valenced pseudowords were used). Thus, these studies suggest that auditory emotional stimuli affect visual processing. A pending issue, though, is the automaticity accompanying such an effect and whether the effect carries over onto metaphorical mapping (see above).

### Automaticity of the Metaphorical Mapping of Emotions onto Space

Some researchers have found that the mapping of visually presented emotion words onto vertical space seem to occur automatically even when the experimental task requires a shallow processing of such mapping; however, such a finding is not clear-cut. As Brookshire et al. (2010) argued, finding vertical space-valence congruity depends on contextual modulation such that the effect disappears with repetition (Experiment 1) and reappears with attention orientation (Experiment 2). Studies on the metaphorical mapping of emotion words on the horizontal (left–right) plane have also found that explicit attention to the valence of the words activates space-valence associations (de la Vega et al., 2012). Thus, these authors argued that an association between horizontal space and valence is not automatic and occurs only when explicit valence assessment is required.

Few studies tapping the effect of auditorily valenced stimuli on visual processing have dealt with the automaticity of this process. A study in which emotional pseudowords were listened to prior to the localisation of a rightward- or leftward-presented dot on the screen indicated that visual spatial cuing by auditory emotional stimuli seems to happen at the very early stages of processing (i.e., between 130 and 190 ms); particularly in the striate visual cortex (Brosch et al., 2009). In this study, visual targets were presented 550 to 750 ms (in increments of 50 ms) after auditory cue onset yet this data was not entered into the statistical analyses. While such SOAs could have been used to further examine the behavioral time-course of the cross-modal audiovisual effect, they were included in the study in order to approximate temporal changes (e.g., variations in stress and pitch) that affect prosody.

It is not known, however, whether such automaticity operates during metaphorical mapping on the vertical space. As mentioned above, some tasks using within-modality visually presented words seem to find a rather automatic mapping from emotions onto space (e.g., Ansorge and Bohner, 2013; see Brookshire et al., 2010 and de la Vega et al., 2012 for examples of studies challenging a clear-cut automatic mapping), but, when longer linguistic units are used, the effect exists only when an explicit emotional evaluation is required (Marmolejo-Ramos et al., 2014). In this line, the systematic manipulation of the time interval between auditory and visual stimuli may provide relevant information about the temporal course of the metaphorical interaction of emotions onto bodily space. Due to the use of different sensory inputs, a crucial benefit of a cross-modal paradigm lies in the possibility of a careful examination of the time intervals between stimuli from total overlapping to long time delays. In a similar manner, the study of De Vega et al. (2013) made use of a dual-task approach to better capture the time course of the embodied interaction between action-related language comprehension and action performance. Interestingly, their results showed reverse effects of interference (with SOAs around 100–200 ms) and facilitation (with a SOA of 350 ms), depending exclusively on the timing between action-related words and motor responses.

### The Present Experiments

The present investigation aimed to study the auditory-visual cross-modal mapping of spoken words onto vertical bodily simulated space. The general method consisted in the prior presentation of a spoken word denoting a positive or negative emotion followed by the display of a visual target whose upper or lower location had to be detected by the participants as soon as possible. It is hypothesized that an interaction between emotion words and the vertical spatial axis may be found in the context of a cross-modal procedure according to a ‘positive-up/negative-down’ embodied metaphor. In particular, this could be owing to a faster detection of upper targets after presenting positive auditory words and lower targets after negative words compared with the other alternative combinations between affective valence and vertical position (i.e., positive-down, negative-up).

The study comprised of two experiments. Experiment 1 was a first attempt to study a possible metaphorical association between emotion and vertical space by means of an auditory to visual cross-modal task. Worth noting, the spoken affective words were passively heard by the participants as they were not required to do any task with these auditory words. Perhaps, this passive procedure was the main reason for the absence of affective and embodied effects found in Experiment 1. For this reason, we decided to introduce a task requiring more active listening of the spoken words than in Experiment 2. Here, two groups of participants carried out different tasks with the auditory words in order to compare an explicit emotion-focused task with a non-emotional activity. In both experiments, the time delay between the auditory and visual stimuli was manipulated in order to explore the temporal course of metaphorical mapping between affect and space.

## Experiment 1

The current experiment examined whether auditory infinitive verbs with an affective valence could modulate the response to a localization task in line with the positive-up, negative-down, vertical spatial metaphor. After playing the auditory files containing the affective words, the participants had to speedily detect the position of a visual target, displayed in either a high or low position on the screen. This task did not require that the auditory stimuli were evaluated in order to test whether mere passive listening could be enough to produce an embodied effect based on a metaphorical conceptual-spatial association.

### Method

#### Participants

Seventeen undergraduate students (12 women and 5 men, *M* = 31.6, SD = 7.9, *age*_range_ = 19–48,) from the *Universidad Nacional de Educación a Distancia* (UNED, Spain) participated in the experiment and received course credits for their participation. The experimental protocol was approved by the Bioethics Committee of the UNED. All of them were native Spanish speakers and reported to have normal or corrected-to-normal vision. Two of the participants were left-handed, and the others were right-handed.

#### Apparatus and Stimuli

The visual stimuli were displayed on 19-inch LCD-LED color monitors with a screen resolution of 1024 × 768 pixels, controlled by microcomputers running E-Prime 2.0 software (Psychology Software Tools, 1996–2002). The auditory words were presented through stereo headphones. The visual targets could be displayed in one of two 11.3 cm × 3.0 cm white (255 RGB) boxes (10.8° × 2.9° of visual angle), presented 8.0 cm (7.6° of visual angle) above and below the center of the screen (center-to-center). The visual targets were printed in black (0 RGB) and presented against a light gray background (192 RGB; “silver” according to the E-Prime color palette). The masks were made up of a 29 × 8 matrix checkerboard of black and gray squares (0 and 192 RGB, respectively).

Forty-eight Spanish infinitive verbs denoting emotional states were used. Half of them referred to positive emotions [e.g., *divertir* (to entertain)] and the other half to negative emotions [e.g., *sufrir* (to suffer); see Data Sheet 1]. The verbs were obtained by converting 48 emotional adjectives from Santiago et al.’s (2012) study into their infinitive verbal tense. Twelve additional verbs were selected for the practice block: six positive and six negative. The infinite verbs were spoken by an expert Spanish-speaking female radio announcer in a neutral voice tone and were digitally recorded in a professional radio studio belonging to the UNED’s audiovisual services.

Mean auditory word duration was 640.2 ms (SD = 137.8 ms; range = 359–932 ms). There was no significant difference between the mean duration of positive (*M* = 622.75; SD = 135.95) and negative words [*M* = 657.67; SD = 134.43; *t*(46) = -0.88; *p* > 0.10]. Additional analyses were conducted to compare the number of letters and the frequency of use (according to LEXESP; Sebastián-Gallés et al., 2000) of the positive and negative words. There were no differences in frequency of use [*t*(46) = 0.01, *p* > 0.10] nor in number of letters [*t*(46) = -0.82, *p* > 0.10] between both samples of words.

#### Procedure and Design

**FIGURE 1:**
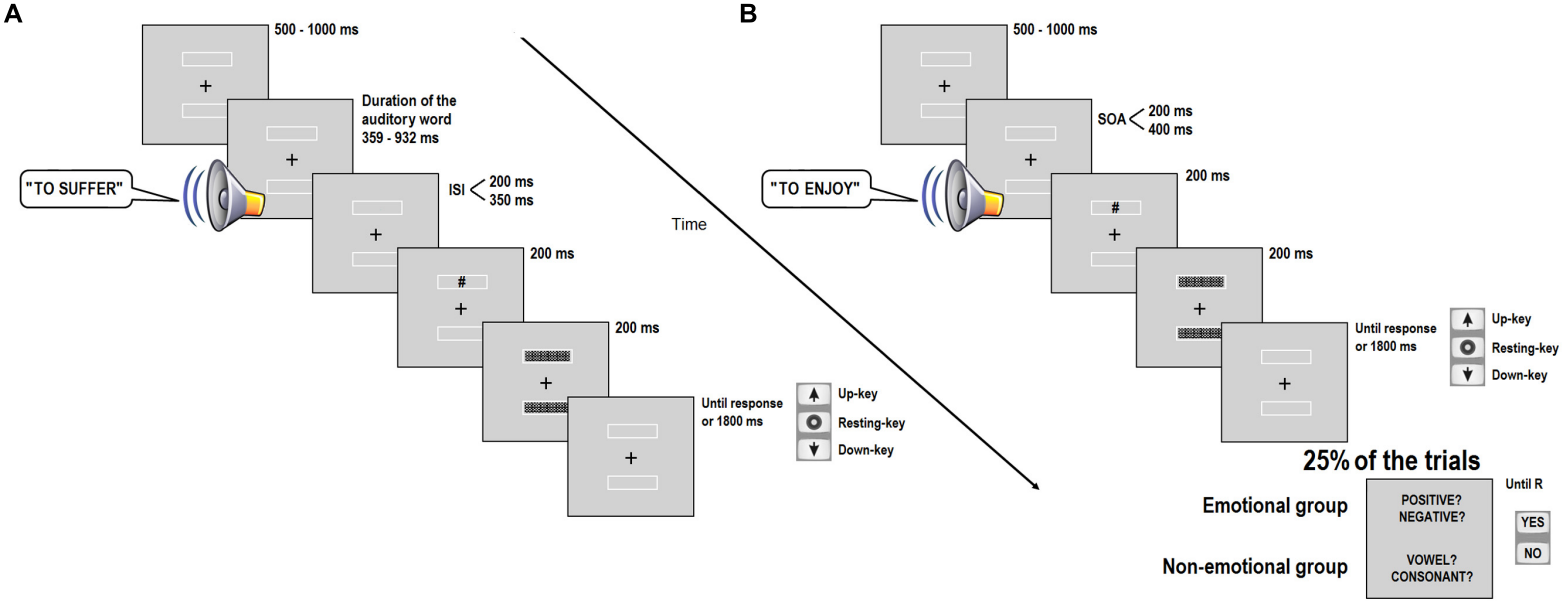
**Illustration of the sequence of events in Experiments 1 **(A)** and 2 (B)**.

Participants were tested individually in a dimly lit, quiet room. The viewing distance was approximately 60 cm. They were instructed to make their responses as quickly as possible while making the minimum number of errors. Each trial started with the presentation of a 1 cm × 1 cm (0.96° of visual angle) cross-shape fixation mark at the center of the screen and two rectangular boxes above and below the fixation. Participants were instructed to remain fixated on the cross until the completion of the trial. After a variable time period oscillating between 500 and 1,000 ms, randomly selected by the program, an auditory word was presented through the headphones. Participants were instructed to passively listen to the auditory word and wait for the presentation of the visual target. At the end of the auditory word, one of two possible inter-stimulus intervals (ISI; 200 or 350 ms) was previously included to the presentation of the visual target, which consisted of a hash sign (#) printed in black (0 RGB). The visual target was displayed for 200 ms in one of the two boxes and, then, two pattern masks filled in the boxes for 200 ms. The target position was determined at random in each trial but ensured an equal proportion of upper and lower trials in the experiment. Participants were instructed to detect (as fast as possible) the position of the target in the vertical axis by indicating whether the hash sign was displayed in the up or down box. The key response procedure was similar to those used by De Vega et al. (2013). The keys “5,” “2,” and “8” from the right-hand side of the keyboard were assigned as the “resting” key, the “up” key and the “down” key, respectively. The iconic arrows printed on the keys “8” (up arrow) and “2” (down arrow) reinforced the spatial interpretation of the response keys in order to simulate a bodily space. The participants placed the index finger of their dominant hand on the “resting” key until they detected the position of the target by pressing “up” or “down” key. After a maximum time of 2,000 ms to respond, the trial was aborted and a message of “no response, try to respond faster” was shown. There was a practice block and six experimental blocks. Each experimental block consisted of 96 trials, for a total of 576 experimental trials, whereas the practice block had 48 trials. Feedback was provided only in the practice trials. The experiment lasted about 40–45 min (see **Figure 1A**). After a short break, an unexpected free-recall test of the spoken words was conducted. A sheet of paper was provided and participants were asked to write down as many words from the experiment as possible for 5 min.

The experimental design included three within-subjects factors: emotional valence of the word (positive vs. negative), visual target position (up vs. down), and ISI (200 vs. 350 ms).

### Results

**FIGURE 2:**
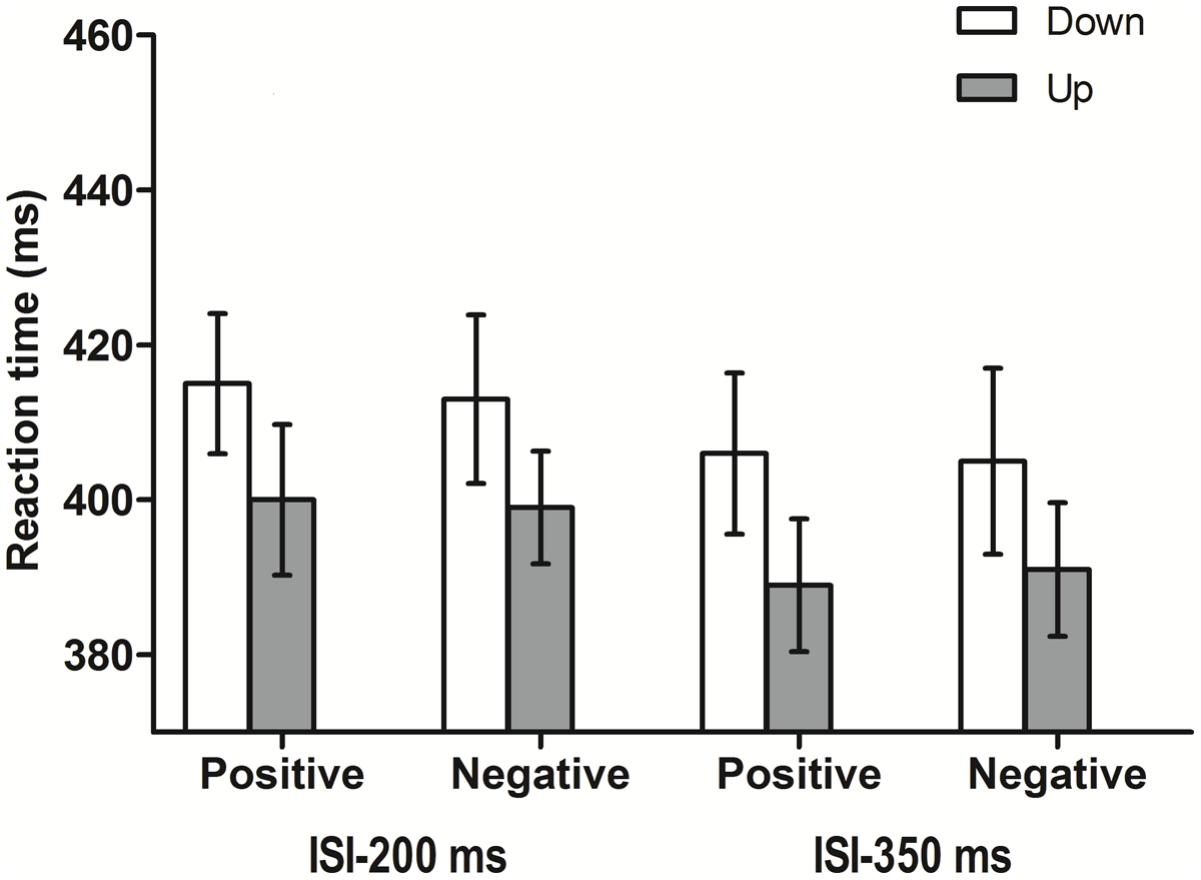
**Means of the median reaction times (ms) for all the conditions in Experiment 1**. Error bars represent 95% CIs adjusted for within-subjects designs (Cousineau, 2005).

Participants responded correctly in 99.5% of all trials (9,741 of 9,792). For the response time (RT) analyses, only correct responses and RTs longer than 200 ms (9,729 of 9,741) were taken into account. The median RT was estimated for each participant in each condition, and these averages were submitted to a parametric ANOVA. The median was chosen since it is an estimator of central tendency robust to outliers (Whelan, 2008). A 2 × 2 × 2 repeated measures ANOVA of the median RTs revealed a main effect of the factor ISI: response were faster (398 ms) after a longer interval between auditory word and visual target compared to shorter interval (407 ms), *F*(1,16) = 18.54, MSE = 15.45, *p* = 0.001, $\eta_{p}^{2}$ = 0.54. Additionally, a marginally significant effect of visual target position was observed, *F*(1,16) = 3.33, MSE = 2327.7, *p* = 0.087, $\eta_{p}^{2}$ = 0.17, suggesting a trend to respond faster to upper positions (395 ms) than to lower locations (410 ms). No other main effects or interactions were significant (see **Figure 2**).

An identical analysis was conducted on accuracy rates. No effects or interactions were significant in this case.

#### Free Recall Test

Missing data for one particular participant was addressed by excluding this particular participant from the analyses. The following analyses included data from the remaining sixteen participants. The global mean number of words recalled was 8.31 (SD = 5.19; range = 1–20), whereas for positive words 4.00 (SD = 2.78) and for negative ones 4.31 (SD = 3.05). The global mean number of words correctly recalled was 6.81 (SD = 4.9); for positive words 3.25 (SD = 2.7) and for negative ones 3.56 (SD = 2.5). On average, participants correctly remembered 14.2% (SD = 10.2) of all the words presented; 13.5% (SD = 11.2) for positive words and 14.8% (10.4) for negative ones. Finally, a conditional proportion correct score was computed for each participant by dividing the number of correct responses by the overall amount of words recalled. The mean conditional correct score was.76 (SD = 0.27) with values oscillating between 0.33 and 1.00.

### Discussion

The results of this experiment indicated there was no effect in the emotional content of the auditory words on the response to the visual target. The unique reliable effect of ISI seems related to a temporal orienting process in a similar manner to the *foreperiod effect* (see Niemi and Näätänen, 1981; Coull, 2009, for reviews). This effect consists in faster RTs for long vs. short intervals between warning signals (e.g., a brief tone) and the imperative stimulus, provided that the different foreperiod durations are equally distributed and randomly presented (Capizzi et al., 2015). In contrast, the task has not been sensitive either to the emotional meaning of the spoken words or the metaphorical link between concepts and space. A possible reason of these null effects might lie in the passive listening induced by the procedure. Participants did not have to apply any cognitive operation involving the auditory word and, apparently, the exclusive role of the auditory words was acting as a preparatory signal. In this line, the relatively poor performance observed in the free recall test suggests that the participants completely ignored the auditory words. According to the results of a previous visual-to-visual experiment of our group (Marmolejo-Ramos et al., 2014), the interaction of emotion with vertical space might require an explicit use in the affective content of the word to obtain a reliable behavioral effect. Other studies displaying single visual tasks (Niedenthal et al., 2009; de la Vega et al., 2012) have reported embodied effects of specific emotions but only in the context of emotion-focused processing tasks. Thus, active listening of the auditory word could lead to a deeper processing of its semantic content and, possibly, promote an embodied interaction with the vertical space.

A simpler explanation for the null embodied effects should not be ruled out. That is, are the auditory words selected representative samples of positive and negative affective stimuli? We adapted the adjectives denoting emotional states that Santiago et al. (2012) included in their experiments to infinitive verbs (see Santiago et al., 2012, p. 1059, for a revision of the method used for the selection of the words). However, we did not confirm that these stimuli were emotionally stimulating for our participants and, therefore it might be possible that the verbs did not represent sufficiently polarized affective values. An explicit evaluation of the emotional valence of each was conducted in Experiment 2 to rule out this possible cause.

Another procedural limitation of Experiment 1 has to do with the interval between the spoken words and the visual target. This interval was introduced as an ISI, that is, the time was counted from the end of the digital file until the onset of the visual target. The marked variability of the files’ duration (range = 359–932 ms; *M* = 640.2 ms; SD = 137.8 ms) might have introduced a confound variable that could make it difficult to stabilize the procedural conditions.

## Experiment 2

The results found in Experiment 1 suggested the possibility of studying a cross-modal embodied effect by means of a different task demanding an active listening of the auditory stimuli, as suggested by previous embodied studies. The current experiment aimed to examine the specific conditions under which the conceptual-physical interaction could emerge. An active listening of the spoken words was introduced by means of two different between-subjects tasks: one requiring an explicit task about the positive or negative affective meaning of auditory stimuli (emotional condition), and another task demanding a mere distinction of the first letter of the word as a vowel or a consonant (non-emotional condition).

If an explicit use of the affective content is needed to produce a semantic-spatial interaction, then an embodied interactive effect restricted to the results from the emotional group should be obtained. In contrast, if a mere active listening of the auditory words is required, then, a conceptual-spatial interaction will also be observed in the results from the non-emotional group. Additionally, a more strict control of the time interval between spoken word and visual target by means of a SOA-procedure (i.e., stimulus onset asynchrony) was used in order to avoid the possibility that the variable duration of the auditory files (from 359 to 932 ms) introduced a disturbing effect. Then, two different time intervals were introduced to the current experiment: 200 and 400 ms. Notice that SOA was measured as time from the onset of the auditory word to the onset of the visual target, causing a partial overlap between both stimuli in most of the trials. However, this procedure does not signify that the visual target appeared before the word was fully processed but only before the word was completely reproduced given that the visual processing requires some time. Thus, it could be assumed that the ending of a process clashes with the beginning of another, making the occurrence of a hypothetical interaction between both cognitive operations easier. Indeed, several classical paradigms inducing semantic interference typically display the target and the distractor/s at the same time, making use of a SOA = 0 ms. Examples of these paradigms are flanker task, parafoveal priming or dichotic listening and all of them are well-known experimental procedures to obtain a consistent effect of semantic interference (see Lachter et al., 2004, for a review).

Besides these improvements, a recognition test and an emotional valence evaluation were included at the end of the experimental session. The inclusion of a recognition test aimed to obtain a more sensible indirect measure of the processing level devoted to the auditory words during the experiment. On the other hand, the emotional valence evaluation was included in the experiment in order to reliably measure the affective salience that the auditory word had in our sample of participants.

### Method

#### Participants

Thirty undergraduate students (twenty-one women and nine men, *M*_age_ = 24.3, SD_age_ = 6.2, *age*_range_ = 19–45,) from the UNED (Spain) participated in the experiment and received course credits for their participation. The experimental protocol was approved by the UNED’s Bioethics Committee. All of the participants were native Spanish speakers and reported to have normal or corrected-to-normal vision. Two of the participants were left-handed, and the others were right-handed. The participants were randomly assigned to two groups of 15 individuals each; an emotional group (*M*_age_ = 24.3, SD_age_ = 3.5), and non-emotional group (*M*_age_ = 24.3, SD_age_ = 8.2).

#### Apparatus and Stimuli

The stimuli and apparatus were identical to those of Experiment 1, with the exception of a new set of 48 infinitive verbs selected as “new” distracter items for the recognition test (24 positive and 24 negative). This new set of infinitive verbs (both positive and negative) were synonyms of, and were matched in length to, those used in Experiment 1.

#### Procedure and Design

The procedure was very similar to Experiment 1 but included several relevant changes. In the current experiment, the temporal interval between the auditory word and the visual target was manipulated by a SOA instead of an ISI, in order to control this variable among the trials, independently of the different duration of the digital audio files. The main task of the participants was the same as Experiment 1, that is, to detect as soon as possible the location of the visual target on the vertical axis. The same keys as Experiment 1 were used (see **Figure 1B**).

A crucial manipulation was related to the different instructions provided for both experimental subgroups. In the emotional group, participants were instructed to carefully listen to the auditory word and judge the emotional valence of the verb as either negative or positive, with the aim of correctly responding to the retrospective question that could be displayed at the end of the trial. In the non-emotional group, participants had to identify whether the first letter of the word was a vowel or a consonant, also with the aim of answering the retrospective question. Retrospective questions were randomly distributed in 25% of the trials so participants could not predict their appearance. Here, a word was displayed in the middle of the screen (e.g., ‘POSITIVE’ for the emotional group or ‘VOWEL’ for the non-emotional group) and the observers had to respond ‘yes’ or ‘no’ by pressing one of two available keys (‘1’ and ‘2’ keys in the top row of numbers with stickers indicating “SÍ”/yes and “NO”) without time response demand and with a different hand than had been used in the main task. There was a practice block and six experimental blocks. Each experimental block consisted of 96 trials, for a total of 576 experimental trials, whereas the practice block had 24 trials. This part of the experiment lasted about 45–50 min.

After the main task was finished, a free-recall task was conducted. The participants had to remember as many auditory words as possible during a 5 min period by writing them on a sheet of paper. Then, participants carried out a computerized recognition task. Ninety-six infinite, positive or negative, verbs (48 ‘old,’ and 48 ‘new’) were randomly displayed on the screen (one word per trial) and participants judged whether the word was heard during the main task by clicking the mouse over the button containing the chosen response (‘YES’ or ‘NO’) without any time constraints. After the presentation of each word, participants had to make a self-paced confidence judgment of their recognition memory. They indicated their confidence in having listened to the presented word by pressing one of eleven response keys from “0” to “10.” A “10” response indicated that they were completely sure of their response, whereas a “0” response indicated that they were completely unsure of the response. Finally, a valence emotional rating task of the 48 auditory words was administered by computer. Participants rated the emotional valence of the words on a 9-point rating scale from -4 (extremely negative) to +4 (extremely positive), considering zero as a neutral value. On the screen, together with the word, nine squares with digits inside from -4 to +4 were displayed on the screen. Participants made their ratings by clicking the mouse over the square containing the chosen number without RT demands.

The experiment resulted in a mixed design with one between-subjects factor (emotional vs. non-emotional groups) and three within-subject factors; emotional valence of the word (positive vs. negative), visual target position (up vs. down), and SOA (200 ms vs. 400 ms).

### Results and Discussion

#### Retrospective Question Task

Performance on the retrospective question trials was high (*M* = 97%; SD = 3.4%; range = 87–100%; emotional group: *M* = 96.87%; SD = 3.6%; non-emotional group: *M* = 97.27%; SD = 3.3%). A one-factor between-subjects ANOVA intended to rule out differences in hit rates between the emotional and non-emotional group showed that there were no differences; *F* < 1.

#### Reaction Times

Regarding the main task, participants responded correctly in 97.6% of all trials (16,870 of 17,280). For the RT analyses, only correct responses and RTs longer than 200 ms (16,828 of 16,870) were considered. As in Experiment 1, the median RT was computed for each participant in each condition, and these averages were submitted to an ANOVA. A 2 × (2 × 2 × 2) mixed ANOVA of the RTs showed main effects of all three within-subjects factors: responses were faster with positive auditory words (429 ms) than with negative words [432 ms; *F*(1,28) = 5.71, MSE = 102.06, *p* = 0.024, $\eta_{p}^{2}$ = 0.17], after a SOA of 400 ms (411 ms) compared with a SOA of 200 ms [449 ms; *F*(1,28) = 95.8, MSE = 897.9, *p* < 0.001, $\eta_{p}^{2}$ = 0.77], and with visual targets displayed in the upper position (419 ms) compared to the lower location [442 ms; *F*(1,28) = 11.54, MSE = 2809.7, *p* = 0.002, $\eta_{p}^{2}$ = 0.29]. Interestingly, there was no main effect of the between-subjects factor group (*F* < 1) showing similar global RTs in both groups. The interaction between group and emotional valence of the word was significant [*F*(1,28) = 5.26, MSE = 537, *p* = 0.03, $\eta_{p}^{2}$ = 0.21], showing that the speeding-up effect of the positive words respect to negative words was reliable in the emotional group (Δ6 ms) but not in the non-emotional group (Δ0 ms). There was also a significant interaction between visual target position and SOA [*F*(1,28) = 13.2, MSE = 220.7, *p* = 0.001, $\eta_{p}^{2}$ = 0.30] suggesting a multiplier effect of the joining together of the longer SOA and the upper position that leads to an even faster response in this condition (396 ms, Δ30 ms compared to longer SOA and lower position) than those in the shorter SOA and upper position (441 ms, Δ16 ms compared to shorter SOA and lower position).

**FIGURE 3:**
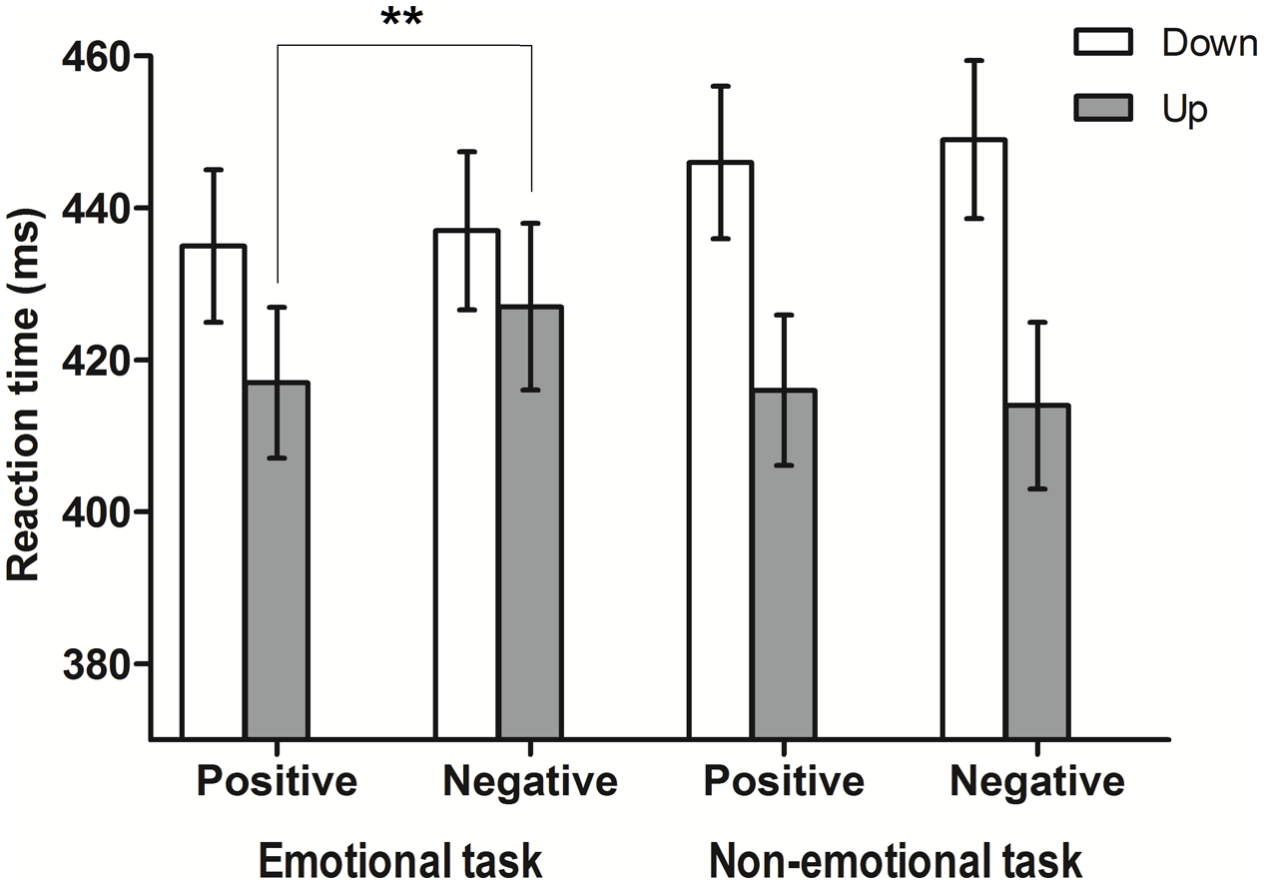
**Means of the median reaction times (ms) for the three-way interaction group, emotional valence, and position in Experiment 2**. Error bars represent 95% CIs adjusted for within-subjects designs (Cousineau, 2005). ^∗∗^*p* < 0.01.

Interestingly, the main effect of the emotional valence of the word, as well as its interaction with the factor group, support a semantic effect of the affective meaning on the response only reliable for the emotional group (which is in contrast with the null effect of this factor in Experiment 1). The effect of SOA replicates the result of the factor ISI of Experiment 1 and may also be based on a temporal orienting effect. Remarkably, the critical effect for the purposes of the present work is a significant three-way interaction between the factors group, emotional valence of the words and visual target position [*F*(1,28) = 4.4, MSE = 114.8, *p* = 0.045, $\eta_{p}^{2}$ = 0.16]. *Post hoc* comparisons using Bonferroni correction showed that the emotional group detected the target faster in the upper position after a positive word (417 ms) compared to a negative one (427 ms; *p* = 0.004). In contrast, no differences in the non-emotional group between those conditions were observed (416 ms vs. 413 ms; *p* = 0.454). No significant effect of the emotional valence of the word on the responses to lower positions was observed, neither in the emotional group (positive word: 435; negative word: 437 ms; *p* = 0.363) nor the non-emotional group (positive word: 446; negative word: 448 ms; *p* = 0.454; see **Figure 3**).

Lastly, a three-way interaction including emotional valence × target position × SOA was significant too [*F*(1,28) = 9.44, MSE = 60.4, *p* = 0.005, $\eta_{p}^{2}$ = 0.25], indicating that the combination of a long SOA, positive valence and upper position generated an even faster response (393 ms) compared with the response to a short SOA, negative valence and upper position (400 ms; *p* = 0.041).

#### Accuracy Rates

Identical analyses were conducted on accuracy rates. The 2 × (2 × 2 × 2) mixed ANOVA of the hit rates only revealed a significant interaction effect between visual target position and SOA [*F*(1,28) = 4.56, MSE < 0.001, *p* < 0.05, $\eta_{p}^{2}$ = 0.14]. However, pair-wise comparisons applying Bonferroni correction did not indicate any significant effect (all *p* > 0.05).

#### Free Recall Test

The mean number of words evoked for the emotional group was 11.94 (SD = 4.11; range = 7–19), whereas, for the non-emotional group 11.4 (SD = 5.17; range = 4–22). The mean number of words correctly evoked for the emotional group was 10.13 (SD = 3.2) and, for the non-emotional group 7.8 (SD = 4.66). On average, participants of the emotional group correctly remembered 21.1% (SD = 6.7) of all the words presented; whereas, the non-emotional group correctly remembered 16.25% (SD = 9.7). A comparison between the group from Experiment 1 and the two groups from the Experiment 2 was carried out by means of a mixed 3 × 2 ANOVA with the factors group and emotional valence of the word, as well as the proportion of items correctly evoked as dependent measure. Neither significant main effects nor interactions between factors reached statistical significance (all *p* > 0.05). A conditional proportion correct score was obtained for each participant. The emotional group obtained an average of.86 (SD = 0.10), whereas the non-emotional group showed an average of 0.67 (SD = 0.23). A one-factor between-subjects ANOVA with the factor group and the conditional correct score as measure suggested differences between the groups [*F*(2,46) = 3.12, *p* = 0.054]. However, *post hoc* comparisons did not detect any significant pair-wise comparisons (all *p* > 0.05).

#### Recognition Test

A direct measure of word recognition (*d*′) was calculated for each participant. The measures were obtained by treating “old” words as signal and “new” words as noise. The individual *d*′ values ranged between 1.18 and 2.54, and the overall mean was 1.65 (SD = 0.19). A mixed 2 × 2 ANOVA with the factors group (emotional vs. non-emotional) and emotional valence of the word and *d*′ values as the dependent variable was conducted. Only the between-subject factor group showed a significant effect [*F*(1,24) = 19.72, *p* < 0.001], in that there was a better recognition rate in the emotional group (*d*′ = 2.02) than in the non-emotional sample (*d*′ = 1.43). Neither a difference between positive and negative words nor an interaction between the two factors was found (both *F* < 1).

To analyse the data from the recognition confidence judgments, a mixed 2 × (2 × 2) ANOVA with the factors group (emotional vs. non-emotional), emotional valence (positive vs. negative) and the type of word (“new” vs. “old”) was conducted on the mean rating of the confidence scores. A significant difference between groups was observed [*F*(1,25) = 6.5, MSE = 3.98, *p* < 0.05, $\eta_{p}^{2}$ = 0.21], in the sense of a higher confidence in the emotional group (*M* = 7.79; SD = 1.01) with respect to the non-emotional group (*M* = 6.81; SD = 0.99). Additionally, the “old” stimuli had significantly more confident judgments (*M* = 7.77; SD = 1.07) than the “new” verbs [*M* = 6.80; SD = 1.25; *F*(1,25) = 41.3, MSE = 0.616, *p* < 0.001, $\eta_{p}^{2}$ = 0.62]. Neither a difference between positive and negative words nor any interaction between the factors was found (all *F* < 1.2).

#### Emotional Valence Evaluation

Mean valence rating for each word was averaged (see Data Sheet 1). The mean rating for the positive words was 2.78 (SD = 0.61) and for the negative ones -2.61 (SD = 0.67), showing a clear polarized difference between them. Two single-sample *t*-tests showed that these mean ratings were significantly different from the neutral score of zero both for positive words [*t*(23) = 22.5, *p* < 0.001] and negative items [*t*(23) = -19.12, *p* < 0.001]. Additionally, a single-sample *t*-test comparing the absolute values (or modulus) of positive and negative words was insignificant, *t*(23) = 0.88, *p* = 0.39, suggesting that both categories of words are polarized to a similar degree. When comparing the ratings provided by both subject groups, more extreme responses were offered by the emotional group than the non-emotional, both for positive words [*M* = 3.00 and *M* = 2.57, respectively; *t*(23) = 6.12, *p* < 0.001] and for negative words [*M* = -2.76 and *M* = -2.49, respectively; *t*(23) = -4.5, *p* < 0.001]. Interestingly, there was no difference in a global comparison of mean valence between both groups including all the words [*t*(47) = 1.19, *p* > 0.10], which suggests that the polarized responses from the emotional group were mutually compensated.

### Discussion

Strikingly, the pattern of RTs supports an interaction between affective activation and the visual vertical axis that is reliable only when the participants were engaged in an emotion-focused task. These results can be interpreted as supporting an association between emotional concepts and the physical vertical axis but only when the instructions demand an explicit decision on the emotional valence of the spoken words or, at least, a processing of the meaning of the word. The absence of this conceptual–spatial mapping in the results from the non-emotional group suggests that an active scrutiny of the words is not enough to generate an embodied interaction. Two possible requirements could be considered in addition to the active listening of the word to obtain an interactive effect between affect and space. On the one hand, a direct handling of the emotional meaning of the words, on the other hand, a deeper level of processing of the information (in terms of Craik and Lockhart, 1972). The results from the recognition task showed a typical level-of-processing effect since a deep processing (i.e., semantic processing) leads to a more robust memory trace as well as higher confidence judgments, while shallow processing (i.e., phonemic or orthographic analysis) results in a more fragile memory and lower confidence in recognition. However, the current design is not qualified to disentangle between both alternatives.

Remarkably, the lack of a main effect of the emotional valence (for the non-emotional group) suggests that the participants may have indeed failed to interpret the meaning of the words. This result contrasts with findings from previous studies that did not ask observers to explicitly judge emotional valence but still found behavioral effects linked to emotionally charged stimuli (e.g., Eastwood et al., 2003; Harris and Pashler, 2004; Estes and Adelman, 2008)^3^. However, it should be taken into account that most of the previous findings have been obtained by visual stimulation (affecting visual processing too; e.g., Harris and Pashler, 2004). In our case, the auditory nature of the affective stimuli especially the cross-modal interaction with another sensory modality could have diminished the usual effects given by other procedures.

Similar to Experiment 1, while the SOA exerted a main effect over the responses based on a temporal preparation, this factor did not modulate the interaction between emotion and spatial axis. Longer intervals between stimuli should be implemented in future experiments to carefully explore the temporal requirements of this conceptual-physical interaction. Regarding this point, an important element of our procedure must be considered, i.e., the retrospective question relating to the spoken word at the end of the sequence of events (in 25% of the trials, strictly speaking). The introduction of such a question forced participants to maintain in working memory (WM) an active representation of the relevant information extracted from the auditory stimuli. This procedure is similar to prior studies showing that the contents of WM can exert an influence over the deployment of attention in visual search tasks arising with asynchronies ranging from 200 to 4000 ms (see Soto et al., 2008, for a revision). Importantly, the WM effects on search were absent when observers were merely exposed to the memory cue without a later report (Soto et al., 2005; Olivers et al., 2006), similar to our Experiment 1. Accordingly, it might be considered that the embodied interaction observed here could be due to the active maintenance in WM of emotionally valenced information irrespective of the specific interval between stimuli (although see Xie et al., 2015). Crucially, this possibility should be taken into account for future research on this topic.

## General Discussion

In the context of within-modal visual tasks, previous studies have reported evidence for an association between emotionally valenced concepts and the vertical as well as horizontal space (e.g., Meier and Robinson, 2004; de la Vega et al., 2012; Santiago et al., 2012; Marmolejo-Ramos et al., 2014; Damjanovic and Santiago, 2015). Other lines of research have studied the influence of emotions activated by other sensory modalities different from visual system (e.g., Tajadura-Jiménez et al., 2010; Rantala et al., 2013) and, even, the interaction between different perceptual modalities during the processing of affective stimuli (e.g., Gerdes et al., 2014; Velasco et al., 2014). Nevertheless, to our knowledge, no previous work has tackled the study of cross-modal interactions between emotionally valenced concepts and bodily space from an embodied standpoint. The current research is the first step toward the study of the metaphorical mapping of emotions onto vertical space by means of an auditory to visual cross-modal paradigm.

Experiment 1 failed to observe such a cross-modal embodiment suggesting that passive listening to the conceptual stimuli was not enough to generate a bias in the detection of the visual target. The participants were not assigned any task related to the spoken words and this absence of cognitive analysis might have led to a null bias of the affective load on the response. Previous studies have provided evidence for the necessary explicit use of the semantic information to observe the embodiment of specific emotions (Niedenthal et al., 2009; de la Vega et al., 2012; Marmolejo-Ramos et al., 2014). On the basis of these findings, we introduced active listening of the spoken words in Experiment 2, by including two different tasks that were applied to different participant samples. Here, the results did show a mapping between emotions induced by auditory words and the vertical space involved in the location detection task of a visual stimulus. This result consisted of a faster detection of the target in the upper position after positive words compared with those after negative words. In contrast, the responses to target displayed at the lower position were not sensible to the different emotional content of the spoken words. This result has similar precedents in previous related research. Recently, Marmolejo-Ramos et al. (2014; Experiment 2) have reported a reliable priming effect of the emotional valence of sentences representing emotional contexts on the processing of visual probes at the upper position, which was not observed for lower positions (see also Xie et al., 2015). In the same direction, the metaphorical congruency effects between affect and vertical space found by Meier and Robinson (2004) and Santiago et al. (2012; see Experiments 1 and 3) showed a higher effect size (in the sense of a higher difference between mean RTs of congruent and incongruent trials) at an upper than lower location; although it is true that the embodied congruency effect was also significant at lower positions, in contrast to our findings. Interestingly, all the three cited studies observed a significant main effect of position, showing global faster responses to targets displayed at upper than lower locations, thus being congruent with our work.

Crucially, the cross-modal embodied interaction found in the present study was restricted to the participants that carried out a semantic emotion-focused analysis of the auditory information. Taken together, the results of our experiments suggest that the association of affective valence and vertical space is not activated automatically during speech processing. However, the exact nature of the task needed to obtain the embodied effect cannot be distinctly established with our experimental design. The emotional group performed a valence-decision task while the other group had to identify the first letter of the spoken word for which emotional content was irrelevant. Notice that the application of an emotional-based criterion was not the exclusive difference due to both between-subjects conditions. That is, an evident divergence regarding the level of processing between a semantic versus a phonemic analysis was presented without a choice to separate them considering the present results. Undoubtedly, this crucial issue should be examined in future research.

The current study is a cross-modal task in that auditory stimuli preceded the presentation of visual items. However, the relation between the auditory and visual stimuli was metaphorical in that, as previous research shows (e.g., Marmolejo-Ramos et al., 2013), emotion concepts can be represented in bodily space. Thus, the task used herein is in fact a cross-modal metaphorical-mapping task. Although the results indicate such mapping seems to occur only when an explicit evaluation of the stimuli is required, it does not exclude that task-dependent factors could lead to different results. It might be the case, for example, that an implicit association did not occur simply because the horizontal (i.e., over the left and right ear) presentation of the auditory stimuli did not facilitate mapping onto the visual vertical plane. Thus, a task in which the location source of the auditory emotional stimuli matches visual vertical spatial locations (see e.g., Spence et al., 2001) could be instrumental in further studying cross-modal metaphorical mapping. By the same token, it would be informative to know whether the mapping holds the other way around. That is, would the cross-modal mapping hold when visual emotional stimuli precede the location of auditory sources onto space? Note that in this study only two sensory modalities are being considered. Hence, the cross-modal processing occurring among these and other modalities (i.e., tactile, olfactory, and gustative) need to be investigated in the context of emotions and metaphorical mapping.

From a theoretical perspective, merely tentative, the current results fit better with the restrained embodiment theories described by Meteyard et al. (2012), such as “secondary embodiment” (sensory and motor system are independent but directly associated) or “weak embodiment” (partially dependence in the relationship to sensory-motor systems). In this line, our results are compatible with an activation of both sensorial and motor systems when a semantic representation is explicitly accessed and opposed to previous findings supporting an automatic, incidental valenced-induced activation of spatial features (e.g., Meier and Robinson, 2004; Gozli et al., 2013; but see Brookshire et al., 2010). Note that the access to the semantic representation may be modulated by the definition of semantic processing and what counts as deeper or more “explicit processes” in comparison to shallow processing required by the task. The distinction between weak and strong theories has also been related to current theories about the embodiment of emotion concepts and explicitly describes how the current findings would confirm balanced/moderate versions of the embodiment hypothesis. In this context, a crucial issue is related to the conceptualization of automaticity. In contrast with the traditional view of automaticity as a dichotomous *all-or-nothing* variable, Brookshire et al. (2010) investigated to what extent is the activation of embodied representations automatic. Remarkably, our procedure could be useful to explore the limits of automaticity in the occurrence of space-valence congruity effects. The inclusion of retrospective measures of memory of the valenced stimuli provide us with an indirect measure of the degree or level of processing devoted to the valenced words, which might be correlated to the effect size of the embodied effects obtained. In Experiment 2, a better recognition of the auditory words in the *emotional group* is compatible with a deeper processing of the valenced stimuli that, at the same time, is correlated with a significant space-valence interaction. However, it is possible that the small sample size in the current study, and hence lack of power, could have hidden this specific kind of embodiment phenomena.

It might be the case that cross-modal metaphorical mapping needs mild to low embodiment and neuropsychological and neuroimaging research could be instrumental in determining the timing and brain localisation of this type of cross-modality (be it real or conceptual). In regards to the localisation, it could be entertained that metaphorical mapping onto bodily space could be processed in the left hemisphere hippocampus as this area is known for dealing with information, mainly linguistic, that feeds into the generation of semantic spaces (see O’Keefe and Nadel, 1979). Indeed, some entorhinal cortex activation could be expected since this area deals with the representation of position, direction, and velocity (Sargolini et al., 2006). In other words, if entorhinal and hippocampal structures aid in the representation of space (see Moser et al., 2008), it is thus tenable that these structures play some role in the representation of metaphorical mappings onto bodily space. We believe that most of the metaphorical processing could be handled by these areas; however, as these areas project to the neocortex, and vice versa via perirhinal and parahippocampal cortex, some mild activation of sensorimotor and somatosensory cortical areas could be observed. This speculation leads us to believe that the processing of cross-modal metaphorical mapping might need mild to low levels of embodiment. Nonetheless, this conjecture is yet to be empirically investigated.

The current work examined the association between affect and vertical space by using a cross-modal procedure. The auditory stimuli selected for our study were spoken words denoting an emotion. Interestingly, for future research, it might be relevant to include emotion sounds (e.g., grunts, sighs, screams) or even to manipulate the prosody of the spoken words in an affective fashion. Such a novelty would provide us with a more direct test of the cross-modal interactions between affect and spatial location^4^. An important advantage of our procedure is that it allows manipulation, in a completely independent manner, of the timing of the visual and auditory stimulation in order to explore the temporal requirements of a metaphorical mapping between emotion and bodily space. The visual and auditory stimuli can be displayed simultaneously or with different SOAs or ISIs. Another potential innovation would be the introduction of a dichotic listening procedure in order to manipulate the extent of cognitive resources devoted to the auditory items. Undoubtedly, such a procedural improvement will serve as an important step in the study of the role of attention, level of processing, and the limits of automaticity in the occurrence of interactive effects between affect and bodily space.

## Conclusion

Our study is a first step toward the study of a cross-modal metaphorical mapping of emotions onto vertical space. The results obtained show that (i) a cross-modal association of affective valence and vertical space is possible but that (ii) this embodied association is not activated automatically because (iii) an explicit evaluation of the emotionally valenced words is needed to observe an interaction between emotion concepts and bodily simulated space.

## Conflict of Interest Statement

The authors declare that the research was conducted in the absence of any commercial or financial relationships that could be construed as a potential conflict of interest.

## Footnotes

1 We thank one of the reviewers for suggesting this possible explanation.

2 It is worth clarifying at this point that in Table 1 in Gerdes et al. (2014), the study by Harrison and Davies (2013, p. 5) is perhaps mistakenly cited as an example of no influence of emotional sounds on a visual task but in the text this mistake is not present.

3 We thank one of the reviewers for suggesting this point.

4 We thank one of the reviewers for suggesting this interesting innovation for future research.
