# Supplementary material for: Cross-modal metaphorical mapping of spoken emotion words onto vertical space
Source: Front Psychol. 2015 Aug 11;6:1205. doi: 10.3389/fpsyg.2015.01205 (PMC4531208; doi:10.3389/fpsyg.2015.01205)
Supplement: Supplementary file 1 [file Data_Sheet_1.DOC]

**Appendix A: Auditory infinitive verbs denoting emotional states used in the present study**

| Positive words | | | Negative words | | |
| --- | --- | --- | --- | --- | --- |
| Spanish word | English translation | Mean valence rating (SD) | Spanish word | English translation | Mean valence rating (SD) |
| alegrar | to cheer up | 3.33 (0.77) | abandonar | to abandon | -2.74 (1.20) |
| amar | to love | 3.63 (0.82) | abrumar | to overwhelm | -1.41 (1.23) |
| animar | to motivate | 2.70 (0.94) | aburrir | to bore | -1.74 (1.07) |
| apasionar | to excite | 3.26 (0.84) | acomplejar | to inhibit | -3.04 (0.79) |
| bromear | to joke | 1.67 (1.12) | acongojar | to distress | -2.56 (1.03) |
| cantar | to sing | 2.04 (1.04) | agredir | to attack | -3.37 (0.91) |
| confiar | to trust | 2.93 (1.18) | amenazar | to threaten | -3.19 (0.77) |
| dinamizar | to revitalize | 1.89 (1.34) | angustiar | to anguish | -3.11 (0.87) |
| disfrutar | to enjoy | 3.30 (0.71) | apenar | to sadden | -2.15 (0.59) |
| divertir | to entertain | 3.11 (0.92) | asustar | to scare | -2.15 (0.70) |
| enamorar | to seduce | 3.52 (0.74) | avergonzar | to shame | -2.48 (1.10) |
| encantar | to love | 2.89 (0.96) | decepcionar | to disappoint | -3.26 (0.80) |
| entusiasmar | to thrill | 2.96 (0.92) | deprimir | to depress | -3.15 (1.01) |
| felicitar | to congratulate | 2.44 (1.20) | desconfiar | to distrust | -2.56 (1.10) |
| gozar | to enjoy | 3.48 (0.63) | desesperar | to despair | -2.85 (0.85) |
| ilusionar | to excite | 2.70 (1.18) | desilusionar | to disillusion | -2.96 (0.79) |
| premiar | to award | 2.30 (0.94) | dudar | to doubt | -0.74 (0.89) |
| querer | to want | 3.52 (0.63) | enfermar | to sicken | -3.00 (1.15) |
| reír | to laugh | 3.63 (0.62) | frustrar | to frustrate | -2.96 (0.84) |
| relajar | to relax | 2.04 (1.23) | llorar | to cry | -2.04 (1.40) |
| satisfacer | to satisfy | 2.70 (1.15) | preocupar | to worry | -2.26 (1.24) |
| serenar | to calm | 1.96 (1.00) | sufrir | to suffer | -3.52 (0.74) |
| simpatizar | to get on well | 2.44 (0.99) | suspender | to fail | -3.19 (0.98) |
| tranquilizar | to reassure | 2.37 (1.06) | temer | to fear | -2.37 (1.09) |

*Note:* Mean valence ratings and standard deviations (SD) were obtained from the emotional valence evaluation task included in the Experiment 2.
