# Supplementary material for: Cross-modal metaphorical mapping of spoken emotion words onto vertical space
Source: Front Psychol. 2015 Aug 11;6:1205. doi: 10.3389/fpsyg.2015.01205 (PMC4531208; doi:10.3389/fpsyg.2015.01205)
Supplement: Supplementary file 2 [file Data_Sheet_2.DOC]

**Appendix B: Synonyms of the auditory words used as distractors in the recognition test of the Experiment 2**

| Positive synonyms | | Negative synonyms | |
| --- | --- | --- | --- |
| Spanish word | English translation | Spanish word | English translation |
| activar | to stimulate | abochornar | to embarrass |
| alentar | to encourage | abusar | to abuse |
| amenizar | to liven up | afligir | to distress |
| bailar | to dance | alarmar | to alarm |
| brindar | to dedicate | atracar | to mug |
| congeniar | to get on well | cansar | to tire |
| congratular | to congratulate | contrariar | to upset |
| conseguir | to achieve | desalentar | to discourage |
| contentar | to please | desencantar | to disillusion |
| deleitar | to delight | desengañar | to disabuse |
| descansar | to rest | desertar | to desert |
| desear | to wish | desolar | to devastate |
| embelesar | to charm | espantar | to appal |
| embriagar | to intoxicate | fracasar | to fail |
| excitar | to excite | golpear | to hit |
| festejar | to celebrate | hundir | to demoralize |
| integrar | to integrate | incomodar | to bother |
| mejorar | to improve | irritar | to irritate |
| motivar | to motivate | mortificar | to mortify |
| recompensar | to reward | quebrantar | to undermine |
| regocijar | to delight | recelar | to suspect |
| seducir | to seduce | sollozar | to sob |
| sosegar | to calm | temblar | to tremble |
| suavizar | to soften | titubear | to hesitate |
